# Expression Analysis of Mitochondrial Energy Metabolism−Related Genes Identifies IRS2 as a Key Modulator in M2 Synovial Macrophages of Osteoarthritis

**DOI:** 10.3390/biomedicines14071493

**Published:** 2026-06-30

**Authors:** Yunlong Yang, Nianlong Zhang, Xuyang Li, Enbei Xie, Yangyu Wu, Jianlin Zhou

**Affiliations:** 1 Department of Orthopedics, Renmin Hospital of Wuhan University, No. 99 Zhangzhidong Road, Wuchang District, Wuhan 430060, China; 2019305232053@whu.edu.cn (Y.Y.); 13341270376@163.com (N.Z.); rmlixuyang@163.com (X.L.); 2020305233082@whu.edu.cn (E.X.); 17762530201@126.com (Y.W.); 2 Department of Orthopedics, the Seventh Hospital of Wuhan, Wuhan 430060, China

**Keywords:** osteoarthritis, synovium, immune infiltration, single−cell RNA−seq analysis, mitochondrial energy metabolism, macrophage polarization

## Abstract

**Background**: Mitochondrial bioenergetic dysregulation disrupts immune−metabolic homeostasis and promotes pro−inflammatory microenvironments in osteoarthritis (OA) synovitis. However, the mechanistic contributions of mitochondrial energy metabolism to synovitis pathogenesis in OA remain poorly defined. **Methods**: We analyzed mitochondrial energy metabolism−related genes (*MEMRGs*) in OA synovitis by integrating transcriptomic data from OA synovial tissues (GSE55235, GSE55457). LASSO regression and maximal clique centrality (MCC) algorithms were applied to identify hub genes, and single−cell RNA sequencing (GSE152805) was used to examine cell−type−specific expression patterns. Functional validation was performed in IRS2−knockdown THP−1 macrophages. **Results**: We identified 22 mitochondrial energy metabolism−related differentially expressed genes (MEMR−DEGs), which were enriched in the AMPK signaling, glucagon signaling, and insulin signaling pathways. Four hub genes (*FOXO3*, *FASN*, *PTGS2*, *IRS2*) were identified, and their expression was negatively correlated with synovial macrophage infiltration. Single−cell RNA sequencing revealed that *IRS2* was specifically upregulated in a synovial macrophage cluster. Functional studies in IRS2−knockdown THP−1 macrophages demonstrated that IRS2 deficiency impaired IL−4−induced M2 macrophage polarization and reduced mitochondrial membrane potential and ATP synthesis, which was mediated by the suppression of the AKT/FOXO1 signaling. **Conclusions**: IRS2 potentially influences mitochondrial energy metabolism, as evidenced by the maintenance of mitochondrial membrane potential and ATP synthesis, via the AKT/FOXO1 signaling pathways to maintain synovial macrophage M2 polarization homeostasis. These findings provide novel molecular targets for addressing immune−metabolic pathways in OA therapy.

## 1. Introduction

Osteoarthritis (OA) is a chronic degenerative disease characterized by the key pathological features of articular cartilage degeneration, synovitis, and osteophyte formation. It is recognized by the World Health Organization as the seventh leading cause of disability in the global elderly population [1,2]. Epidemiological data reveal that over 528 million individuals globally (approximately 7% of the global population) are affected by OA, which is clinically characterized by persistent joint pain and functional mobility impairment. These manifestations not only markedly diminish patients’ quality of life but also impose a substantial socioeconomic burden [3]. Recent studies have demonstrated that synovial inflammation serves not only as an early pathological marker of OA but also as a critical driver of disease progression to advanced stages [4,5]. At the molecular level, inflammatory mediators such as IL−1β and PGE2 accelerate the pathological degradation of the cartilage extracellular matrix by activating matrix metalloproteinase (MMP) pathways [6]. Histopathological analyses have revealed that macrophages and T lymphocytes dominate the synovial immune cell infiltrate in OA patients across all disease stages [7,8]. Notably, the substantial infiltration of macrophages and their polarization toward the pro−inflammatory M1 phenotype exacerbate synovial hyperplasia and osteophyte formation via inflammatory cascades [9,10]. Therefore, elucidating the mechanisms governing the dynamic evolution of synovitis and targeting the polarization homeostasis of synovial macrophages may represent novel strategies for halting OA progression.

Emerging evidence suggests that macrophage polarization is fundamentally driven by metabolic reprogramming, with mitochondrial energy metabolism serving as a decisive molecular foundation for immune functional remodeling [11]. Mitochondria, as cellular energy hubs, regulate energy metabolism through the tricarboxylic acid (TCA) cycle, oxidative phosphorylation (OXPHOS), and fatty acid β−oxidation (FAO), generating substantial ATP via ATP synthase (complex V) [12]. Evidence shows that energy metabolism plays a pivotal role in regulating inflammatory responses and immune cell functionality while dynamically modulating their biological activities [13]. M1 macrophages exhibit enhanced glycolysis and lactate production to meet their bioenergetic demands [14,15], whereas M2 macrophages depend predominantly on mitochondrial oxidative metabolism, encompassing FAO, TCA, and OXPHOS [16]. Significantly, the natural compound songorine ameliorates cartilage damage and synovitis by inducing macrophage metabolic reprogramming, specifically suppressing glycolysis and promoting mitochondrial OXPHOS [17]. These findings implicate mitochondrial metabolism−related genes (MEMRGs) as potential regulatory hubs in OA synovitis.

Insulin receptor substrate 2 (IRS2), a key mediator of insulin signaling, plays an essential role in mitochondrial DNA and protein synthesis, thereby enhancing mitochondrial oxidative capacity and ATP production [18,19]. In the liver tissue of Irs1/Irs2 double−knockout mice, disrupted respiratory chain complexes (III and IV), reduced NAD+/NADH ratios, diminished ATP synthesis, and elevated FOXO1 expression have been observed [20]. FOXO1, a pivotal downstream target of the AKT pathway, serves as a nuclear transcription factor regulating diverse cellular processes via phosphorylation−dependent mechanisms, including differentiation, metabolism, inflammation, and macrophage polarization [21].

We designed this study to investigate the expression profiles of MEMRGs in OA synovial cell clusters and identify key MEMRGs regulating synovial macrophage polarization. By analyzing bulk RNA−seq data, we characterized the expression landscape of hub MEMRGs in the OA synovium and their potential interaction with immune cells. Notably, scRNA−seq analysis revealed that IRS2 expression was specifically enriched in the synovial macrophage cluster compared to other cell populations. Given that the IRS2/AKT has been previously identified as a mechanism regulating M2 polarization in obesity and metabolic diseases [22], this study was designed to explore whether this mechanism plays a central role in the immune microenvironment of chronic synovitis in OA. At the mechanistic level, we demonstrated that IRS2 potentially influences mitochondrial energy metabolism via the AKT/FOXO1 signaling pathways to maintain synovial macrophage M2 polarization homeostasis. This discovery not only highlights the pivotal role of IRS2 in synovial macrophage phenotypic regulation but also provides a potential molecular target for OA therapeutic strategies.

## 2. Materials and Methods

### 2.1. Clinical Samples

Synovial tissues were collected from 5 OA patients (3 females, 2 males; age 56–78 years; Kellgren−Lawrence [K−L] grade 3–4) undergoing total knee arthroplasty and 5 healthy controls (3 females, 2 males; age 36–44 years; K−L grade 0–1). The healthy control synovial tissues were obtained from patients undergoing surgical treatment for tibial plateau fractures caused by severe acute trauma. None of these patients had any prior history of joint diseases or metabolic disorders. Patients with rheumatoid arthritis, metabolic disorders, or infectious joint diseases were excluded. Tissue samples were immediately fixed in precooled 4% paraformaldehyde within 30 min post−surgery. The study was approved by the Ethics Committee of Renmin Hospital Affiliated to Wuhan University on 4 April 2025. All participants provided written informed consent.

### 2.2. Cell Culture and Transfection

THP−1 monocytes (Procell, Wuhan, China) were cultured in RPMI−1640 medium supplemented with 10% fetal bovine serum and 1% penicillin−streptomycin at 37 °C under 5% CO_2_. For differentiation, the cells were seeded at 1 × 10^5^ cells/well and treated with 100 ng/mL phorbol 12−myristate 13−acetate (PMA, 24 h) to generate M0 macrophages. Cells were transfected with IRS2 siRNA (50 pmol per well; see Table S1 for sequences) or a scrambled negative control siRNA (siRNA−NC, designated as ‘Control’ in subsequent functional experiments) using CALNP™ RNAi reagent (D−Nano Therapeutics, Beijing, China). To elucidate the mechanisms underlying the maintenance of anti−inflammatory homeostasis in the synovium, the transfected cells were stimulated with IL−4 and IL−13 (20 ng/mL, 48 h) to induce M2 macrophage polarization. Additionally, to assess the impact of IRS2 deficiency on pro−inflammatory responses, parallel groups of transfected M0 macrophages were stimulated with lipopolysaccharide (LPS, 100 ng/mL, 48 h) to induce M1 macrophage polarization. The siRNA sequences are provided in Supplementary Table S1.

### 2.3. Gene Expression Profiling Data Acquisition and Processing

Synovial bulk RNA−seq datasets GSE55235 (including 10 healthy control samples and 10 OA samples) and GSE55457 (including 10 healthy control samples and 10 OA samples) were downloaded from the Gene Expression Omnibus (GEO) database as the training set. Additionally, the GSE12021 dataset was downloaded as an independent external validation set. Raw CEL files from the GeneChip Human Genome U133 Array (GPL96) platform were processed in R version 4.3.1. Expression values were log2−transformed, and probes were reannotated using platform−specific annotation files (GPL96). For genes mapped to multiple probes, the expression levels were averaged. Batch effect correction and data standardization were performed with the “SVA” package (version 3.38.0) [23]. Principal Component Analysis (PCA) was performed before and after the correction procedure. A comprehensive list of 599 mitochondrial energy metabolism−related genes (MEMRGs) was obtained from the validated gene signature established by Zewei Zhang et al. [24] for our subsequent bioinformatic analysis.

### 2.4. Differentially Expressed Gene Identification

Differentially expressed genes (DEGs) between the OA and healthy controls were identified via the “limma” package (version 3.46.0) [25]. Significance thresholds were strictly set at an adjusted *p*−value (FDR) < 0.05 and |log2Fold change (log2FC)| > 1 to minimize false discovery rates. Volcano plots and hierarchical clustering heatmaps were generated using the “ggplot2” package (version 3.3.3) and the “ComplexHeatmap” package (version 2.6.2), respectively. Overlapping DEGs and mitochondrial energy metabolism−related genes (MEMRGs) were analyzed with the “VennDiagram” package (version 1.6.20), which identified 22 intersecting genes (MEMRG−DEGs).

### 2.5. GO and KEGG Enrichment Analysis

Functional enrichment of the MEMRG−DEGs was performed using the “clusterProfiler” package (version 3.18.1) [26]. Gene Ontology (GO) terms, including Biological Process (BP), Cellular Component (CC), and Molecular Function (MF), along with Kyoto Encyclopedia of Genes and Genomes (KEGG) [27] pathways, were analyzed with a significance threshold of *p* values < 0.05. The top 10 significant terms, ranked by their enrichment ratio, were visualized using the “ggplot2” package.

### 2.6. Identification of Hub MEMRG−DEGs

A protein–protein interaction (PPI) network was constructed using the STRING database (https://string-db.org/) (accessed on 13 April 2025) [28]. The network was then visualized and analyzed with Cytoscape software (version 3.8.2) [29]. Hub genes were ranked using the Maximal Clique Centrality (MCC) algorithm in the CytoHubba plugin [30]. To reinforce feature selection robustness, a parallel Least Absolute Shrinkage and Selection Operator (LASSO) regression analysis was conducted using the “glmnet” package (version 4.1−1). Finally, the integration of the MCC rankings and LASSO regression outputs identified four hub MEMRG−DEGs.

### 2.7. Validation of Hub MEMRG−DEGs Expression and Evaluation of Predictive Biomarker Potential

To evaluate the predictive biomarker potential of the four identified hub MEMRG−DEGs (*FASN*, *FOXO3*, *IRS2*, *PTGS2*), their expression levels were extracted and compared between the OA patients and healthy controls in both the training set and the external validation dataset (GSE12021). Furthermore, Receiver Operating Characteristic (ROC) curves were generated to evaluate the diagnostic accuracy of each hub gene in distinguishing OA from healthy samples. The Area Under the Curve (AUC) was calculated using the “pROC” package (version 1.17.0.1) in R, with an AUC value closer to 1 indicating superior predictive performance.

### 2.8. Single−Sample Gene Set Enrichment Analysis

Single−sample gene−set enrichment analysis (ssGSEA) was performed with the “GSVA” package (version 1.38.2) [31] to quantify immune cell infiltration differences between the OA patients and healthy controls. Statistical correlations between hub genes and immune cell subsets were analyzed using the rcorr function. To robustly control for false positives across multiple comparisons, the resulting *p*−values were adjusted using the Benjamini–Hochberg (BH) method. The significance criteria were strictly set at an adjusted *p*−value (FDR) < 0.05. Heatmaps visualized the correlations between the hub MEMRG−DEGs and immune cells, while boxplots compared the immune cell enrichment scores between OA patients and healthy controls.

### 2.9. Single−Cell Transcriptomic Data Analysis

The single−cell RNA−seq data from the GSE152805 dataset was preprocessed using the “Seurat” package (version 4.0.6) [32]. Low−quality cells were filtered based on the following thresholds: number of detected genes < 200 or >6000 and mitochondrial gene ratio > 20%. Data normalization and scaling were performed to identify the top 2000 highly variable genes. Batch effects were mitigated using the “harmony” package (version 0.1.0) [33] before performing principal component analysis (PCA) for dimensionality reduction and t−distributed stochastic neighbor embedding (t−SNE) for cluster visualization. Cluster−specific differentially expressed genes were identified with the Seurat::FindAllMarkers() function(min.pct = 0.25, logfc.threshold = 0.25). Cells were categorized into 8 distinct cell−type clusters based on acknowledged cell markers: synovial intimal fibroblasts (SIF)−*PRG4*, synovial subintimal fibroblasts (SSF)−*WISP2*, macrophages−*CD68*, smooth muscle cells (SMC)−*RGS5*, endothelial cells (EC)−*TM4SF1*, dendritic cells (DC)−*FCER1A*, mast cells−*TPSAB1*, and proliferative immune cells (ProIC)−*BIRC5* [34]. Gene activity scores for each subpopulation were computed using the “AUCell” package (version 1.12.0) [35].

### 2.10. Western Blot (WB)

Total proteins were specifically extracted from the cultured THP−1 cells using RIPA lysis buffer containing PMSF and protease/phosphatase inhibitors, followed by concentration normalization via the Bicinchoninic Acid (BCA) method. Protein samples were denatured at 99 °C for 10 min and electrophoresed on 10% or 12% SDS–PAGE gels. Separated proteins were transferred to PVDF membranes (Bio−Rad, Hercules, CA, USA, 1704273), which were then blocked with 5% nonfat dry milk for 2 h at 25 °C. Membranes were incubated overnight at 4 °C with the following primary antibodies:IRS2 (Proteintech, Wuhan, China, 20702−1−AP), CD86(Proteintech, 31449−1−AP), pan−AKT antibody (Proteintech, 10176−2−AP), pan−phospho−Akt (Ser−473)(Proteintech, 28731−1−AP), FOXO1 (Affinity, Changzhou, China, AF6416), phospho−FoxO1 (Ser−256) (Affinity, AF3417), ARG1 (Proteintech, 16001−1−AP)(utilized here as an established marker for M2 macrophage polarization), and GAPDH (Proteintech, 10494−1−AP). After three washes with TBST, the membranes were incubated with HRP−conjugated secondary antibodies for 1 h. Protein bands were visualized using an enhanced chemiluminescence (ECL) substrate (Epizyme, Shanghai, China, SQ202), and band intensities were quantified via ImageJ software (version 1.53t), with GAPDH serving as the loading control for normalization.

### 2.11. Immunofluorescence (IF) Staining

Synovial tissues were fixed in 4% paraformaldehyde, paraffin−embedded, and sectioned into 5−μm slices using a microtome. Sections were deparaffinized with a xylene gradient and rehydrated through an ethanol gradient, followed by three 5 min PBS washes. Heat−induced epitope retrieval was performed in preheated 0.01 M citrate buffer for 20 min. To eliminate endogenous peroxidase interference, the sections were incubated with 3% H_2_O_2_ under light−shielded conditions for 25 min. Non−specific binding was blocked with 5% bovine serum albumin (BSA) in PBS for 1 h at room temperature. Sections were then incubated overnight with anti−IRS2 (Abcam, Cambridge, UK, 1:100, ab134101) and anti−CD206 (Abcam, Cambridge, UK, 1:200, ab300621). After washing with PBS, the fluorescence−conjugated secondary antibodies were applied for 1 h at room temperature. Nuclei were counterstained with DAPI, and the sections were mounted with an antifade mounting medium. Imaging was performed using a fluorescence microscope (Olympus, Tokyo, Japan).

### 2.12. Measurement of Mitochondrial Membrane Potential

Mitochondrial membrane potential (ΔΨm) was measured in the in vitro THP−1 cell line model using the JC−1 MitoMP Detection Kit (Dojindo Molecular Technologies, Tokyo, Japan). THP−1 cells were washed with PBS, and then incubated with the JC−1 working solution at 37 °C for 20 min under light−protected conditions. After two washes with JC−1 assay buffer, the cells were imaged using an inverted fluorescence microscope. Red fluorescence indicated intact ΔΨm, whereas green fluorescence signified mitochondrial membrane depolarization.

### 2.13. Measurement of Intracellular ATP Concentration

Intracellular ATP levels were quantified using a commercial ATP Assay Kit according to the manufacturer’s instructions. Briefly, cultured THP−1 cells from different experimental groups were lysed, and the supernatants were collected following centrifugation (12,000× *g*, 4 °C, 5 min). The ATP concentration in the lysates was determined by measuring the appropriate signal using a microplate reader (BioTek Instruments, Winooski, VT, USA). To ensure accurate normalization, the total protein concentration of each corresponding sample was simultaneously measured using a BCA Protein Assay Kit (Proteintech, Wuhan, China). The final intracellular ATP levels were normalized to the total protein content and expressed as μmol/gprot.

### 2.14. Statistical Analysis

Experiments were conducted with three independent biological replicates per group. Analyses were performed in GraphPad Prism 8.0. The normality and homogeneity of variance were verified for all datasets prior to statistical testing. Group differences were analyzed using an unpaired Student’s *t*−test for comparisons between two groups. For datasets involving multiple groups with a single independent variable, a one−way analysis of variance (ANOVA) was utilized. Crucially, for experiments involving two independent variables, a two−way ANOVA was strictly applied. Significance levels were defined as *p* < 0.05 (*), *p* < 0.01 (**), *p* < 0.001 (***), and *p* < 0.0001(****). Where appropriate, particularly in high−throughput differential gene expression and multi−variable correlation analyses, the *p*−values were adjusted using the Benjamini–Hochberg false discovery rate (FDR) method to rigorously control for multiple testing.

## 3. Results

### 3.1. Identification and Functional Enrichment of MEMRG−DEGs

**Figure 1:**
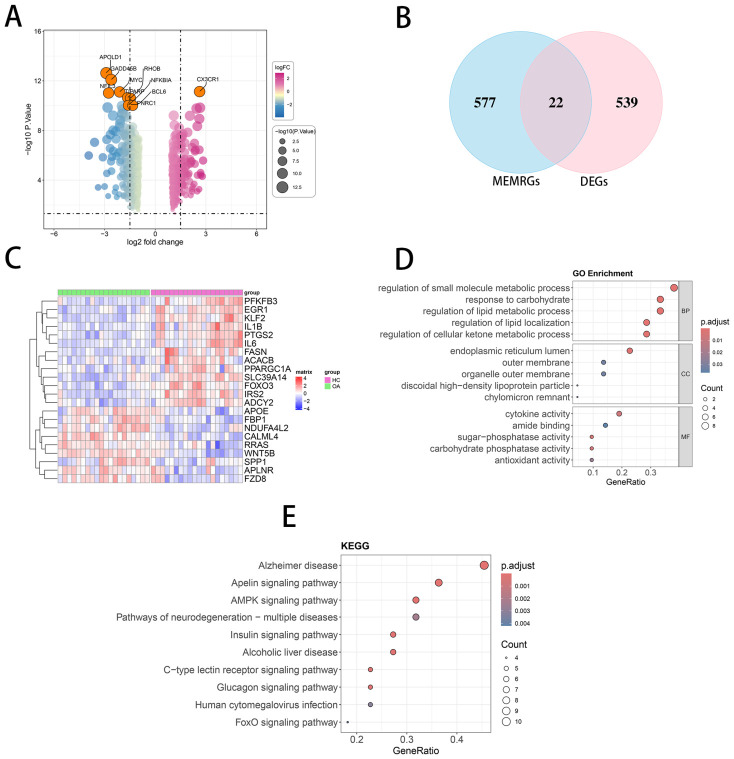
Identification and enrichment analysis of MEMRG-DEGs. (**A**) Volcano plot of DEGs between OA patients and healthy controls. Red and blue dots represent significantly upregulated or downregulated genes in OA synovium. (**B**) Venn diagram of overlaps between DEGs and MEMRGs. (**C**) Heatmap of 22 MEMRG-DEGs in OA synovium. Red and purple hues indicate high or low gene expression. (**D**) Gene Ontology enrichment analysis of MEMRG-DEGs. (**E**) KEGG pathway enrichment analysis of MEMRG-DEGs.

We integrated the GSE55235 and GSE55457 datasets and performed global batch correction prior to downstream analyses. PCA was used to verify the successful removal of batch effects, demonstrating that the samples were clustered by their true biological status rather than dataset origin (Supplementary Figure S1A,B). Following this, differential expression gene (DEG) analysis of MEMRGs identified 297 upregulated and 264 downregulated genes in the OA patients compared to the healthy controls (Figure 1A). A Venn diagram was employed to identify the MEMRGs among the DEGs (Figure 1B). The heatmap confirmed that these MEMRG−DEGs effectively distinguished the OA (*n* = 20) samples from the healthy controls (HC; *n* = 20) (Figure 1C). GO and KEGG enrichment analyses were performed to elucidate the regulatory pathways and biological functions of the MEMRG−DEGs. GO enrichment analysis found that within the Biological Processes (BPs), the MEMRG−DEGs were predominantly enriched in small−molecule metabolic processes, lipid metabolism regulation, and cellular ketone metabolic regulation. For Cellular Components (CCs), the MEMRG−DEGs clustered in the endoplasmic reticulum lumen, the organelle outer membrane, and the outer membrane. In Molecular Functions (MFs), the MEMRG−DEGs were mainly enriched in cytokine activity and amide binding (Figure 1D). KEGG pathway analysis demonstrated significant enrichment of the MEMRG−DEGs in the AMPK, glucagon, insulin, and FOXO signaling pathways (Figure 1E), which regulate macrophage polarization through mitochondrial energy metabolism modulation [11].

### 3.2. Protein–Protein Interaction Network and Hub Gene Screens

**Figure 2:**
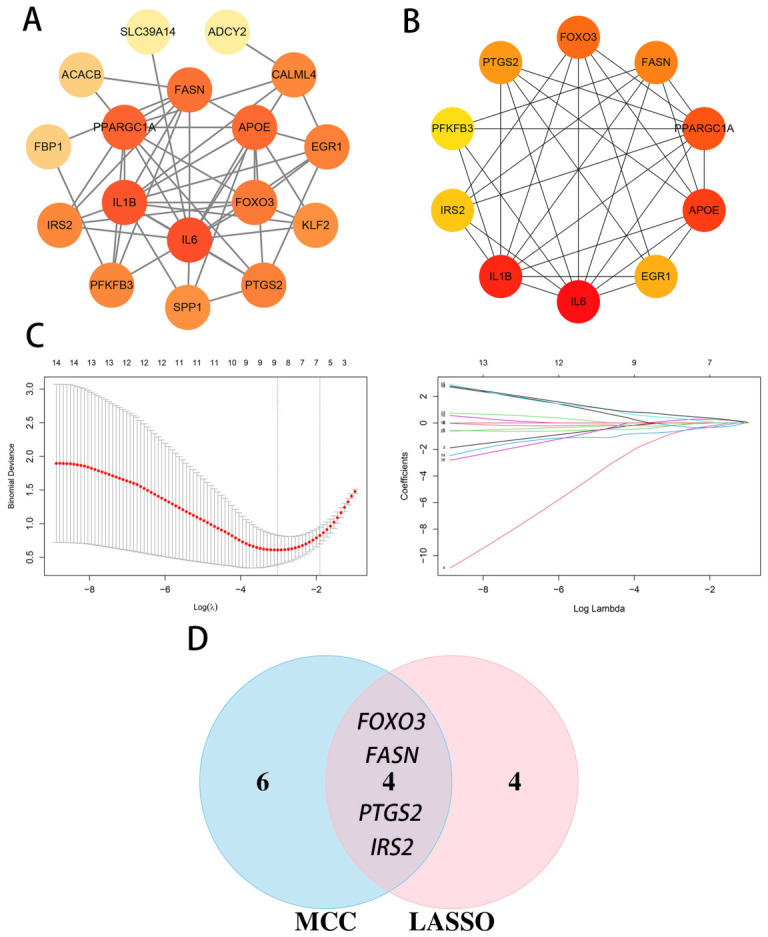
Selection and analysis of hub MEMRG-DEGs. (**A**) The PPI network of the MEMRG-DEGs. Node color intensity correlated with degree values. (**B**) Top 10 hub genes were identified by CytoHubba-MCC. (**C**) Eight hub MEMRG-DEGs were screened by Lasso regression method. (**D**) Venn diagram showed overlapping hub genes from LASSO and MCC methods.

The PPI network of the MEMRG−DEGs was constructed using Cytoscape, and sorted by the degree algorithm. It contained 17 nodes and 50 edges (Figure 2A). The top 10 hub genes were prioritized via the MCC algorithm in the CytoHubba plugin, including *IL6*, *IL1B*, *APOE*, *PPARGC1A*, *FOXO3*, *FASN*, *PTGS2*, *EGR1*, *IRS2*, and *PFKFB3* (Figure 2B). To refine specificity, LASSO regression analysis was performed and eight eigengenes were identified: *FOXO3*, *FASN*, *IRS2*, *CALML4*, *KLF2*, *NDUFA4L2*, *WNT5B*, and *PTGS2* (Figure 2C). Intersection analysis between the MCC algorithm-derived hub genes and the LASSO-selected eigengenes (Figure 2D) revealed four key mitochondrial metabolism-related hub genes: *FOXO3*, *FASN*, *PTGS2*, and *IRS2*.

### 3.3. scRNA-Seq Identified Localization of Hub MEMRG-DEGs

**Figure 3:**
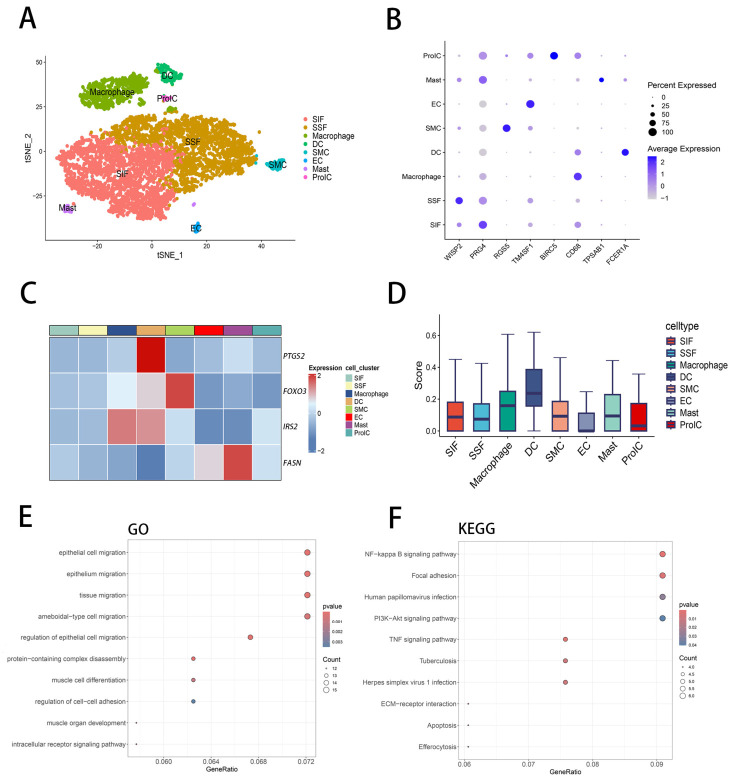
scRNA-seq revealed the localization of hub MEMRG-DEGs. (**A**) Eight cell clusters were identified and visualized using a t-SNE plot. (**B**) Expression of representative marker genes of eight cell clusters. (**C**) The heatmap displayed the expression of four hub MEMRG-DEGs for each cell cluster. (**D**) AUCell scores for four hub MEMRG-DEGs in each cell cluster. (**E**) GO enrichment analysis of upregulated genes in the IRS2-high expression group. (**F**) KEGG enrichment analysis of upregulated genes in the IRS2-low expression group.

To determine the distribution of the four hub MEMRG-DEGs, the scRNA-seq dataset GSE152805 was analyzed using Seurat (resolution = 1.0). Cell clusters were annotated into eight populations based on canonical markers: synovial intimal fibroblasts (SIF), synovial subintimal fibroblasts (SSF), macrophages, smooth muscle cells (SMC), endothelial cells (EC), dendritic cells (DC), mast cells, and proliferative immune cells (ProIC) (Figure 3A,B).Given the core effector roles of fibroblasts and macrophages in OA pathogenesis, subsequent in-depth investigations were specifically focused on these two cellular subpopulations [36]. Gene expression profiling revealed low abundance of *PTGS2*, *FOXO3*, *IRS2*, and *FASN* in the fibroblast subsets (SSF and SIF). In macrophages, *FOXO3* exhibited moderate expression levels, while *IRS2* showed significantly higher expression. Conversely, *PTGS2* and *FASN* remained at low baseline levels (Figure 3C).To quantify mitochondrial energy metabolism activity in the OA patients, the “AUCell” package was employed to analyze functional activity based on the four hub MEMRG-DEGs. Cells with high AUC scores predominantly localized to macrophages and DCs (Figure 3D). To investigate the specific mechanisms of IRS2 in macrophages, we meticulously divided the macrophage subpopulation into high-expression and low-expression groups based on the expression levels of the IRS2 gene, and carried out a systematic enrichment analysis of the differentially expressed genes between these two groups. The results revealed a distinct functional segregation. In the high-expression group, the upregulated genes were primarily enriched in biological processes related to epithelial cell migration, tissue migration, and the regulation of epithelial cell migration, which significantly reflected the tissue-repairing properties of these cells (Figure 3E). Conversely, in the low-expression group, the upregulated genes were mainly enriched in the NF-κB signaling pathway, focal adhesion, and the PI3K-Akt signaling pathway, distinctly demonstrating the pro-inflammatory characteristics of the IRS2-low cells (Figure 3F). These findings indicate that IRS2 expression within synovial macrophages is intrinsically associated with an anti-inflammatory, tissue-repairing phenotype.

### 3.4. Expression Validation and Evaluation of Predictive Biomarker Potential for Hub MEMRG-DEGs

**Figure 4:**
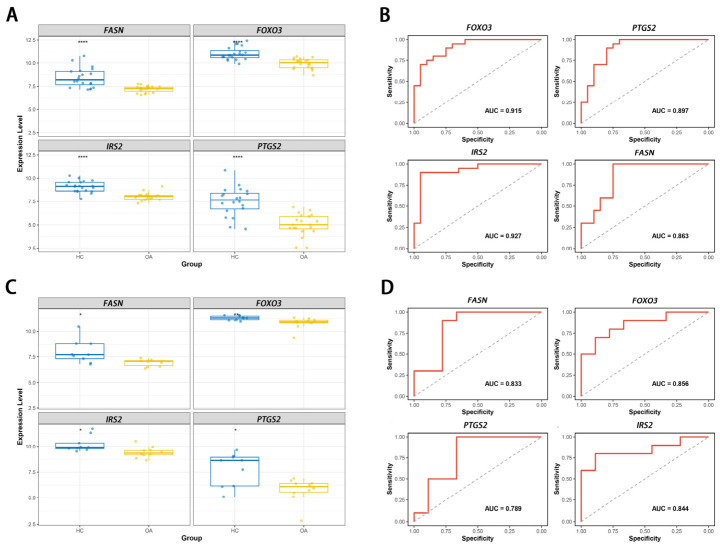
Validation of expression levels and evaluation of predictive biomarker potential for the hub genes. (**A**) Box plots showing the differential expression levels of the four hub genes between the healthy control (HC) and OA groups in the training set. (**B**) ROC curves evaluating the diagnostic accuracy of the four hub genes in the training set. (**C**) Validation of the expression levels of the four hub genes between HC and OA samples in the external validation dataset (GSE12021). (**D**) ROC curves verifying the predictive biomarker potential of the four hub genes in the external validation dataset (GSE12021). Significance levels were defined as *p* < 0.05 (*), *p* < 0.01 (**), and *p* < 0.0001 (****).

We analyzed the expression levels and predictive biomarker potential of the four hub MEMRG-DEGs (*FASN*, *FOXO3*, *IRS2*, *PTGS2*) in the training set. The results revealed that, compared to healthy controls (HC), these four genes were significantly downregulated in the OA samples (Figure 4A). ROC curve analysis demonstrated their remarkably high diagnostic accuracy for OA (all AUCs > 0.85): *IRS2* (0.927), *FOXO3* (0.915), *PTGS2* (0.897), and *FASN* (0.863) (Figure 4B). Subsequently, we validated these findings in an external dataset (GSE12021). Consistent with the training set, these four genes also exhibited significantly lower expression in OA samples (Figure 4C) and maintained robust predictive biomarker potential, with AUC values of *FOXO3* (0.856), *IRS2* (0.844), *FASN* (0.833), and *PTGS2* (0.789) (Figure 4D). In conclusion, *FASN*, *FOXO3*, *IRS2*, and *PTGS2* are significantly downregulated in OA and exhibit robust predictive capability as potential biomarkers.

### 3.5. IRS2 Demonstrated Significant Correlation with Macrophages

**Figure 5:**
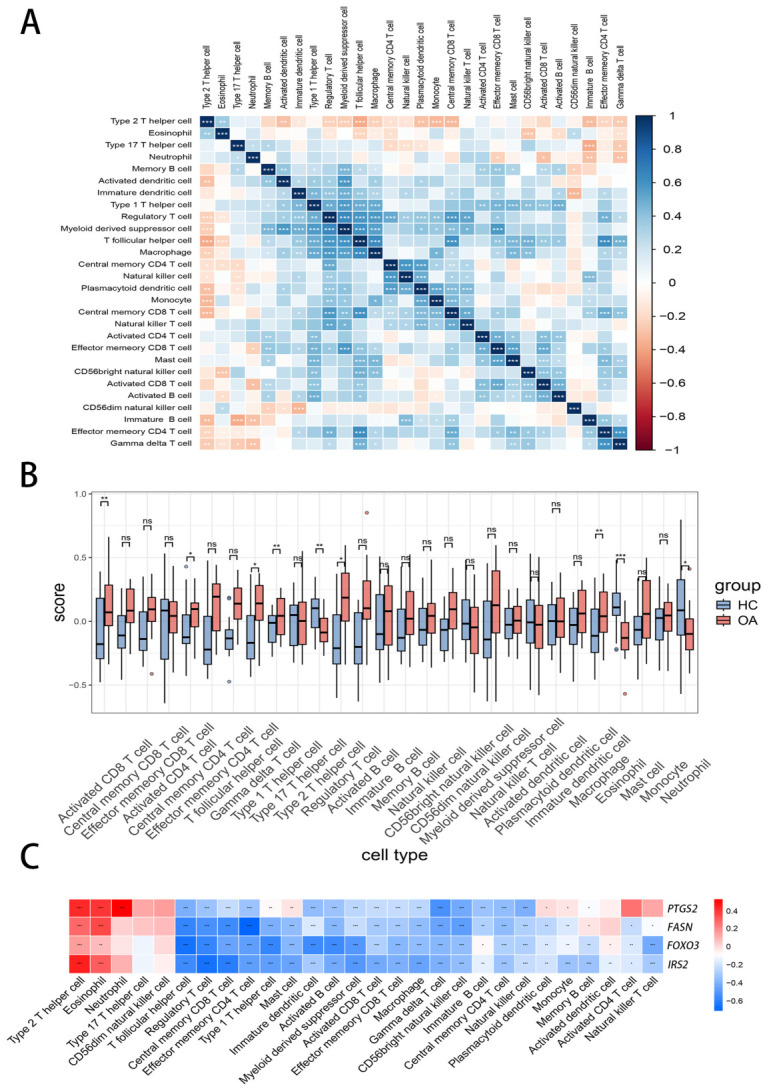
Correlation between hub MEMRG-DEGs and immune infiltration in OA. (**A**) Correlation matrix of 28 immune cell types in OA synovium. (**B**) 28 types of immune cells between OA patients and healthy controls by ssGSEA. (**C**) The correlation between hub MEMRG-DEGs and immune cells. Significance levels were defined as ns: not significant, *p* < 0.05 (*), *p* < 0.01 (**), *p* < 0.001 (***).

To explore the immune cell infiltration characteristics in the OA patients, single-sample gene set enrichment analysis (ssGSEA) was performed to compare immune infiltration scores between the healthy controls and OA patients. Immune cell population correlation analysis revealed that macrophages exhibited significant positive correlations with Type 1 T helper cells and regulatory T cells. In contrast, negative correlations were observed with Type 2 T helper cells (Figure 5A). ssGSEA profiling revealed widespread alterations in the immune infiltration landscape of OA patients, involving activated CD8+ T cells, Th1/Th2 cells, macrophages, and eosinophils (Figure 5B).Notably, OA appears to exert differential, opposing effects on the T helper cell subsets. While Th1 cell infiltration is significantly increased, Th2 cells exhibit a distinct downward trend. The significance of these differential effects lies in the potential Th1/Th2 imbalance within the OA microenvironment. This opposing dynamic suggests a distinct shift towards a pro-inflammatory state. Consequently, Type 1 T helper cells and macrophages are closely associated with OA pathogenesis and may synergistically contribute to synovial inflammation [37,38]. Furthermore, correlation analysis demonstrated significant negative correlations between I*RS2*, *FOXO3*, *FASN*, and *PTGS2* expression levels and the infiltration levels of Th1 cells and macrophages (Figure 5C). Among these genes, *IRS2* demonstrated the strongest inverse correlation with macrophage infiltration.

### 3.6. IRS2 Was Downregulated in OA Synovium

**Figure 6:**
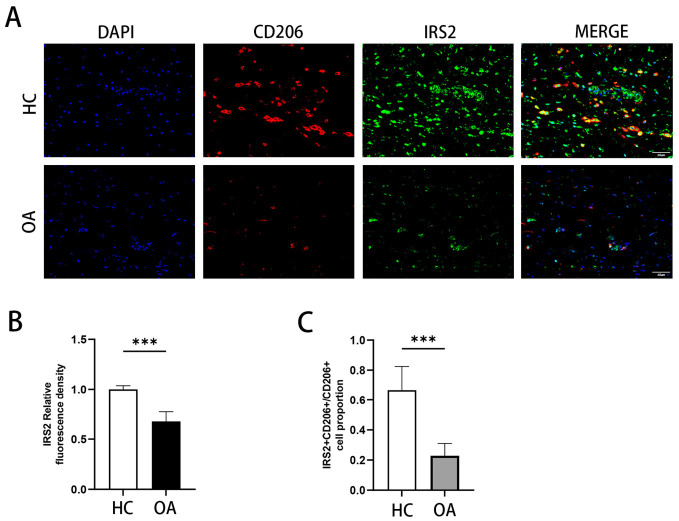
IRS2 was downregulated in OA synovium. (**A**) Immunofluorescence (IF) staining of IRS2 in control and OA synovium. (**B**) Quantification of IRS2 fluorescence intensity. (**C**) Colocalization analysis of IRS2 and CD206 in M2-type macrophages. Significance levels were defined as *p* < 0.001 (***).

**Figure 7:**
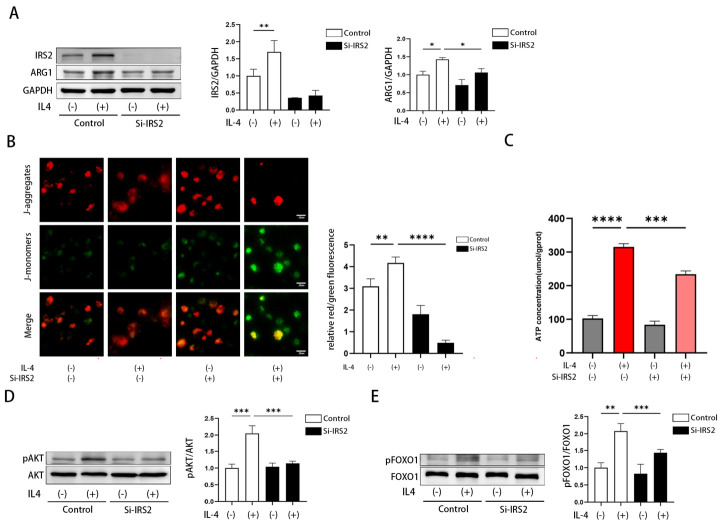
IRS2 knockdown impaired M2 macrophage polarization and mitochondrial function via AKT/FOXO1 inhibition. (**A**) Expression of M2 macrophage marker ARG1 in IRS2-knockdown THP-1 macrophages. (**B**) JC-1 fluorescence ratios reflected mitochondrial membrane potential. (**C**) Intracellular ATP concentration in control and IRS2-knockdown THP-1 macrophages following IL-4 stimulation. (**D**) AKT phosphorylation and protein levels in the THP-1 macrophages transfected with scrambled negative control siRNA (Control) or IRS2 siRNA (si-IRS2) after IL-4 stimulation. (**E**) FOXO1 phosphorylation and protein levels in the THP-1 macrophages transfected with scrambled negative control siRNA (Control) or IRS2 siRNA (si-IRS2) after IL-4 stimulation. Significance levels were defined as *p* < 0.05 (*), *p* < 0.01 (**), *p* < 0.001 (***), *p* < 0.0001 (****).

To assess the association between IRS2 expression and OA, immunofluorescence staining was performed on synovial tissues from the OA patients and healthy controls (HC). Quantitative analysis revealed a significant reduction in IRS2 expression and M2 macrophage marker CD206 in the OA tissues compared with the HC (Figure 6A,B). Notably, IRS2 exhibited robust spatial co-localization with CD206 in the healthy controls, indicating predominant IRS2 expression in physiological M2 macrophages. In contrast, this co-localization pattern was significantly attenuated in the OA synovial tissues (Figure 6C). Interestingly, Western blot analysis demonstrated that IL-4 stimulation significantly upregulated IRS2 protein expression in the THP-1-derived macrophages (Figure 7A). This apparent paradox likely stems from the differing cytokine milieus. The progressive OA synovium is a pro-inflammatory microenvironment inherently lacking sufficient M2-inducing signals (e.g., IL-4), which likely suppresses IRS2 expression in vivo. Conversely, direct IL-4 stimulation in vitro forcefully drives M2 polarization, thereby strongly upregulating IRS2.

### 3.7. Downregulation of IRS2 Impaired M2 Macrophage Polarization

Finally, we found that IRS2 plays a pivotal role in the IL-4-induced M2 polarization of macrophages. IRS2 was knocked down via small interfering RNA (Figure S2 in Supplementary Files). A Western blot assay showed that IRS2 knockdown diminished IL-4-induced ARG1 activity (Figure 7A). We quantified the JC-1 fluorescence ratio to assess the mitochondrial membrane potential (MMP). Compared with the control group, IL-4 stimulation slightly elevated the MMP, as evidenced by an increased red fluorescence intensity. However, the IRS2-knockdown macrophages (IL-4 + si-IRS2) exhibited a significantly reduced red/green fluorescence ratio compared to the IL-4 control group, indicating a decrease in the MMP and subsequent mitochondrial membrane depolarization (Figure 7B). Furthermore, consistent with the loss of mitochondrial membrane potential, IRS2 knockdown drastically reduced the IL-4-induced intracellular ATP concentration, indicating a severe impairment in mitochondrial bioenergetics (Figure 7C). These data implicate IRS2 as a critical regulator of M2-type macrophage polarization and mitochondrial functional homeostasis. To further ascertain the impact of IRS2 deficiency on the broader inflammatory balance, we assessed its role in M1 polarization. Notably, Western blot analysis revealed that IRS2 knockdown significantly exacerbated the LPS-induced expression of the M1 marker CD86 (Figure S3 in Supplementary Files). This demonstrates that the loss of IRS2 not only impairs anti-inflammatory M2 polarization but also actively amplifies pro-inflammatory M1 responses. Mechanistically, IRS2 knockdown suppressed the phosphorylation of AKT at Ser473 and downstream transcription factor FOXO1 phosphorylation at Ser256 in response to IL-4 (Figure 7D,E).These findings demonstrate that IRS2 may regulate mitochondrial energy metabolism by activating the AKT/FOXO1 signaling pathway, which is required for M2 macrophage polarization. This molecular mechanism gives new insights into aberrant macrophage polarization in inflammatory diseases such as osteoarthritis.

## 4. Discussion

Synovitis, driven by the aberrant activation and phenotypic polarization of synovial macrophages, is recognized as a critical accelerator of OA progression [39]. Emerging evidence highlights metabolic reprogramming as a fundamental driver of this macrophage plasticity [11]. Mitochondrial energy metabolism not only dictates cellular bioenergetics but also tightly orchestrates inflammatory cascades through ROS generation and metabolic shifts [40]. In the OA microenvironment, this metabolic imbalance severely impairs the protective, anti-inflammatory M2 macrophage phenotype, thereby failing to counterbalance inflammatory mediators and exacerbating joint destruction [41,42]. In the present study, we aimed to decode the regulatory network of MEMRGs in OA synovitis. By integrating multi-omics datasets with in vitro functional validations, we identified IRS2 as a potential regulator linking mitochondrial function and macrophage polarization—a discovery that provides potential new perspectives for targeted OA therapy.

This study identified 22 MEMRG-DEGs through the integrated analysis of RNA sequencing data from the GEO database. Functional enrichment analysis highlighted the regulation of small molecule metabolic processes and lipid metabolic processes as the core enriched modules. Notably, these pathways are intrinsically linked to mitochondrial energy metabolism. Emerging evidence underscores that mitochondrial energy metabolism dysregulation plays a pivotal role in OA pathogenesis [43]. Specifically, dysfunction of the mitochondrial electron transport chain reduces ATP synthesis, induces oxidative mtDNA damage, and promotes excessive ROS accumulation, thereby forming a vicious cycle of mitochondrial dysfunction and oxidative stress [44]. Further studies have shown that this disorder disrupts the balance between pro-inflammatory and anti-inflammatory factors in the synovial tissue and significantly enhances the levels of chemokines (MCP-1,MIP-1α), thus amplifying inflammatory signaling and exacerbating OA joint inflammation [45,46].

Next, the combined application of the LASSO regression and MCC algorithms identified 4 hub genes from the 22 MEMRG-DEGs: *FOXO3*, *FASN*, *PTGS2*, and *IRS2*. Biologically, these genes are deeply embedded in the regulation of synovial inflammation and macrophage metabolic reprogramming. For instance, FOXO3 serves as a crucial transcription factor that mitigates oxidative stress and modulates macrophage survival and polarization. FASN, a key enzyme in de novo lipogenesis, supports the metabolic and lipid demands essential for macrophage inflammatory activation. Additionally, PTGS2 (commonly known as COX-2) is a well-established driver of inflammatory prostaglandin synthesis, profoundly exacerbating synovial inflammation and cartilage destruction. Studies have reported that OA progression is linked to the heightened infiltration of immune cells, particularly macrophages and T cells [47,48]. To assess their immunoregulatory roles, we conducted correlation analyses between these hub genes and immune cell infiltration scores. Quantitative analyses demonstrated significant associations between the four hub MEMRG-DEGs and the predominant infiltrating immune cell subpopulations, particularly macrophages. Functioning as pivotal regulatory elements within the synovial immune microenvironment, macrophages manifest significant phenotypic dysregulation in OA. While much of the existing literature emphasizes the pathogenic role of pro-inflammatory macrophages, the present study focuses on the critical failure of synovial macrophages to maintain a protective, anti-inflammatory M2 phenotype. M2 macrophages are essential, and their polarization is fundamentally driven by metabolic reprogramming, which is particularly reliant on intact mitochondrial oxidative phosphorylation (OXPHOS) [11,49]. When mitochondrial energy metabolism is dysregulated, the substantial bioenergetic demands required for M2 polarization cannot be met. Consequently, rescuing mitochondrial energy metabolism to restore M2 macrophage polarization homeostasis represents a critical frontier for OA therapeutic development.

Analysis of the scRNA-seq data revealed significantly elevated AUCell enrichment scores of the core genes in macrophages. We also observed that the IRS2 protein was highly expressed in macrophages, which strongly suggests that IRS2 mainly plays a role in macrophages. Nevertheless, while our single-cell data directed our specific focus toward synovial macrophages, the regulatory scope of IRS2 extends beyond a single cell lineage. It broadly regulates the survival, metabolic reprogramming, and function of other immune cells, including T lymphocytes and dendritic cells, which collectively shape the complex inflammatory landscape of OA synovitis. In T lymphocytes, IRS2 acts as a crucial intracellular transducer for insulin and various cytokine receptors, orchestrating the PI3K/AKT/mTOR signaling cascade to meet the high bioenergetic demands required for T cell activation, proliferation, and subset differentiation (such as the delicate balance between Th1 and regulatory T cells) [50]. Similarly, IRS-mediated signaling tightly regulates the metabolic shift, maturation, and antigen-presenting capacities of dendritic cells [51]. When IRS2 signaling is broadly suppressed in the OA microenvironment, these diverse immune populations are forced into a metabolic shift that typically favors a pro-inflammatory state. Consequently, the downregulation of IRS2 in the OA synovium likely precipitates a comprehensive breakdown of immune homeostasis not just in macrophages but across the broader immune network, thereby synergistically driving joint inflammation and cartilage degradation.

Subsequent mechanistic investigations therefore focused on IRS2. Compelling evidence from Heller et al. has demonstrated that the type I IL-4R (IL-4Rα/γc) mediates macrophage target gene expression through the specific activation of IRS2 [52]. Building upon this, Kubota et al. established the necessity of the IL-4/IRS2/AKT signaling axis for M2a subtype macrophage differentiation, which worsens metabolic inflammation due to decreased IRS2 expression in a high-fat diet-induced obesity mouse model [21,22]. However, despite its well-documented role in systemic metabolic diseases, it remains entirely unknown whether the disruption of this classic metabolic immune pathway acts as a critical driver of the localized inflammatory microenvironment in human OA synovitis. Our experiments showed that IRS2 knockdown caused a significant decrease in the mitochondrial membrane potential and a drastic reduction in intracellular ATP production in macrophages, suppressing IL-4-induced ARG1 activity. Since M2 macrophages predominantly rely on oxidative phosphorylation (OXPHOS) to meet their high bioenergetic demands, these observations together strongly imply that IRS2 deficiency impairs the OXPHOS machinery. Although we did not directly quantify oxygen consumption rates (OCR) or reactive oxygen species (ROS) in this specific model, extensive literature demonstrates that such mitochondrial depolarization and hampered ATP production are fundamentally linked to electron transport chain dysfunction and subsequent ROS accumulation. Beyond impairing the M2 phenotype, our in vitro data demonstrated that IRS2 knockdown actively exacerbated LPS-induced M1 polarization, as evidenced by significantly upregulated CD86 expression. This confirms that IRS2 deficiency not only cripples the M2 anti-inflammatory counterbalance but also amplifies the pro-inflammatory cascades. Moreover, IRS2 knockdown impaired Akt (Ser-473) phosphorylation and FOXO1 (Ser-256) phosphorylation, aligning with prior studies. The inhibition of the AKT/FOXO1 signaling axis suggests that IRS2 may influence the anti-inflammatory function of macrophages by regulating mitochondrial energy metabolism. This mechanistic insight provides a novel therapeutic strategy for restoring immune microenvironment homeostasis through IRS2-targeted interventions.

Despite providing novel insights into the immune-metabolic mechanisms of OA, several limitations of this study should be acknowledged. First, the scRNA-seq dataset (GSE152805) included only three OA synovial samples without healthy controls, limiting our ability to definitively confirm OA-specific transcriptional changes across all cellular subpopulations. Second, owing to the small sample size (*n* = 5/group) and the lack of rigorous age matching, our tissue samples and microarray data were exclusively obtained from patients with advanced osteoarthritis (Kellgren–Lawrence grades 3–4). Consequently, our findings mainly reflect chronic synovial inflammation; the early acute-phase dynamics of the IRS2-mediated metabolic–immune axis remain unresolved, warranting large-scale, age-matched cohort studies across the full disease continuum. Third, our in vitro mechanistic investigations relied on the THP-1 leukemic cell line. While this reproducible model minimizes inter-patient heterogeneity for initial screening, its intrinsic differences in metabolic and polarization dynamics mean it cannot fully recapitulate the physiological OA microenvironment. Consequently, our mechanistic claims regarding the IRS2/AKT/FOXO1 axis remain preliminary. Future studies must validate these findings using primary human synovial macrophages or patient-derived MDMs to confirm their translational relevance. Finally, the lack of in vivo validation, such as using macrophage-specific Irs2 conditional knockout mice, prevents the direct establishment of system-level causal relationships within the complex joint environment. Future longitudinal, multi-center studies with expanded cohorts, primary human cells, and conditional knockout models are warranted to validate these findings and the dynamic alterations of the AKT/FOXO1 signaling pathway.

## 5. Conclusions

In conclusion, our study successfully integrated transcriptomic screening to identify 22 MEMRG-DEGs and highlighted four hub genes (*FOXO3*, *FASN*, *PTGS2*, and *IRS2*) associated with OA pathology. Furthermore, we elucidated a molecular mechanism wherein IRS2 potentially influences mitochondrial energy metabolism via the AKT/FOXO1 signaling pathway to maintain synovial macrophage M2 polarization homeostasis. These findings provide new predictive biomarkers and therapeutic targets for OA intervention.

## Data Availability

Data derived from public domain resources: The data presented in this study are available in the Gene Expression Omnibus (GEO) database (https://www.ncbi.nlm.nih.gov/geo/). These data were derived from the following resources available in the public domain: GSE55235, https://www.ncbi.nlm.nih.gov/geo/query/acc.cgi?acc=GSE55235, GSE55457, https://www.ncbi.nlm.nih.gov/geo/query/acc.cgi?acc=GSE55457, GSE152805, https://www.ncbi.nlm.nih.gov/geo/query/acc.cgi?acc=GSE152805, GSE12021, https://www.ncbi.nlm.nih.gov/geo/query/acc.cgi?acc=GSE12021, (all the links were accessed on 31 October 2025).

## Supplementary Materials

The following supporting information can be downloaded at: https://www.mdpi.com/article/10.3390/biomedicines14071493/s1, Figure S1. Principal Component Analysis (PCA) plots demonstrating the elimination of batch effects. (A) The PCA score plot before batch correction between datasets GSE55235 and GSE55457. (B) The PCA score plot after batch correction between datasets GSE55235 and GSE55457; Figure S2. Western blot was used to verify the efficiency and specificity of IRS2 knockdown by specific siRNAs compared to the scrambled negative control siRNA (SiRNA-NC); Figure S3. Expression of M1 macrophage markerCD86 in IRS2-knockdown THP-1 macrophages; Table S1. Small interfering RNA sequence of IRS2.
