# Supplementary material for: Expression Analysis of Mitochondrial Energy Metabolism−Related Genes Identifies IRS2 as a Key Modulator in M2 Synovial Macrophages of Osteoarthritis
Source: Biomedicines. 2026 Jun 30;14(7):1493. doi: 10.3390/biomedicines14071493 (PMC13404189; doi:10.3390/biomedicines14071493)
Supplement: Supplementary file 1 [file biomedicines-14-01493-s001.zip › biomedicines-4284821-supplementary.pdf]

Supplementary Material

S1

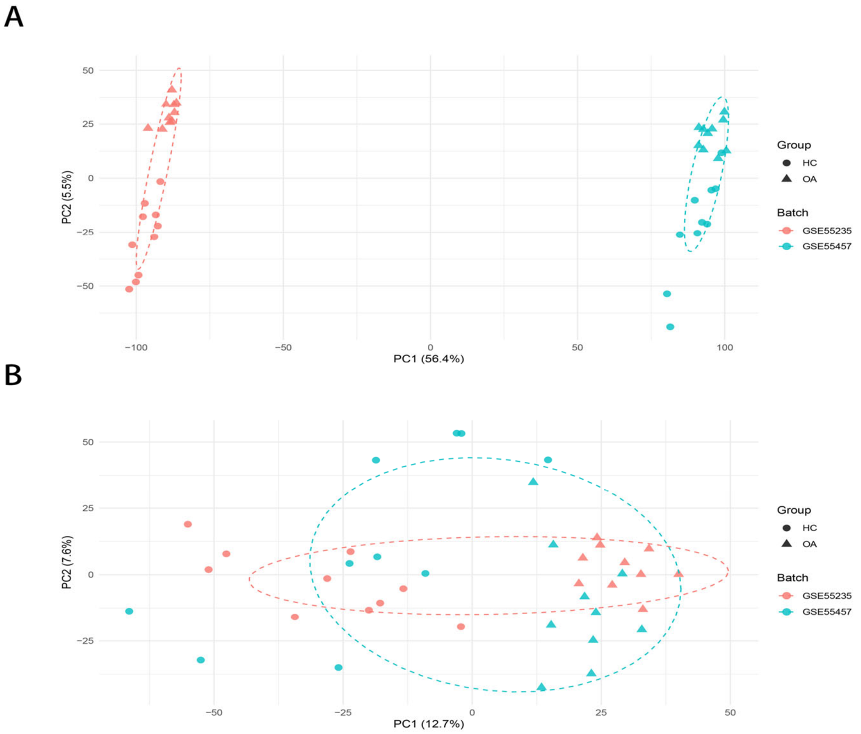

Figure S1. Principal Component Analysis (PCA) plots demonstrating the elimination of batch effects. (A) The PCA score plot before batch correction between datasets GSE55235 and GSE55457. (B) The PCA score plot after batch correction between datasets GSE55235 and GSE55457.

S2

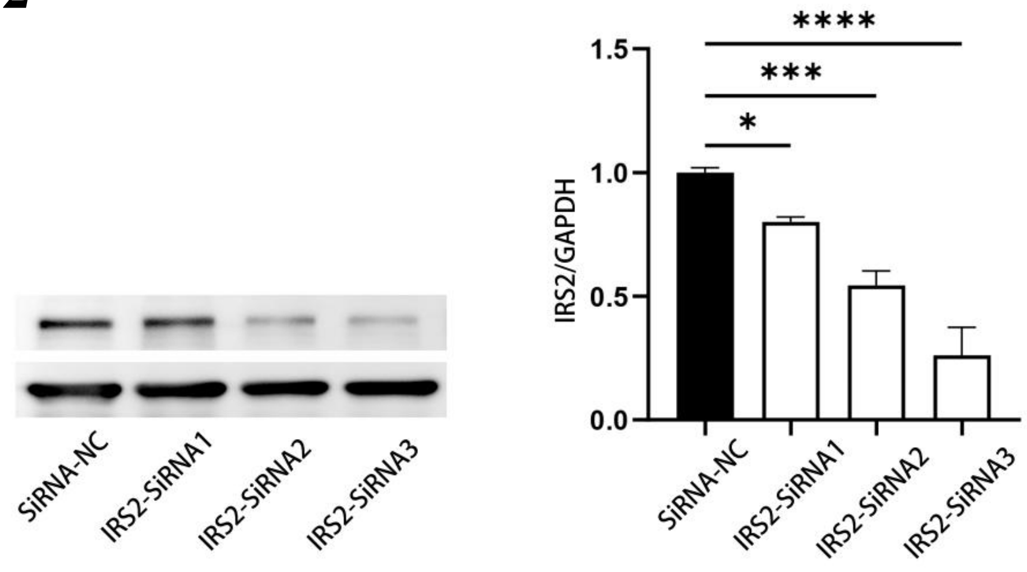

Figure S2 Western blot was used to verify the efficiency and specificity of IRS2 knockdown by specific siRNAs compared to the scrambled negative control siRNA (SiRNA-NC).

S3

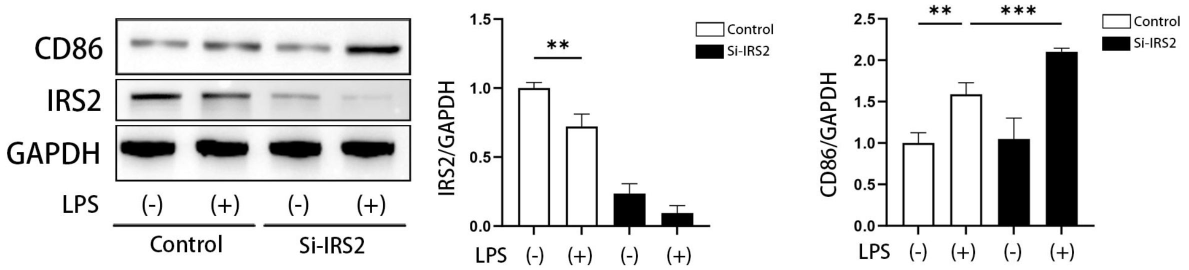

Figure S3: Expression of M1 macrophage markerCD86 in IRS2-knockdown THP-1 macrophages.

Table S1 Small interfering RNA sequence of IRS2

Home-Human	siRNA-sequences	
	sense（5'-3'）	antisense（5'-3'）
IRS2-SiRNA1	CCCAGAGGACUACGGAGACAUTT	AUGUCUCCGUAGUCCUCUGGGTT
IRS2-SiRNA2	CGAGUACAUCAACAUCGACUUTT	AAGUCGAUGUUGAUGUACUCGTT
IRS2-SiRNA3	GCUGGAUGAAUACACCCUGAUTT	AUCAGGGUGUAUUCAUCCAGCTT
